# Bridging Voices Through Food: Sub‐Saharan African Young People's Perspectives of Food and Nutrition Policy in Victoria, Australia

**DOI:** 10.1002/hpja.70234

**Published:** 2026-08-13

**Authors:** Khalid Muse, Ayuba Issaka, Dheepa Jeyapalan, Nicola Miranda, Kevin Kapeke, Kathryn Backholer, Veronica Nunez, Sally Schultz, Hannah Jongebloed, Chioma Anidi, Christina Zorbas

**Affiliations:** ^1^ Institute for Health Transformation, Deakin Centre for Global Preventive Health and Nutrition, Deakin University Geelong Victoria Australia; ^2^ The Victorian Health Promotion Foundation (VicHealth) West Melbourne Victoria Australia; ^3^ Institute for Health Transformation, Centre for Quality and Patient Safety, School of Nursing and Midwifery, Faculty of Health, Deakin University Geelong Australia

**Keywords:** African Australians, food equity, food policy, food security, migrant health, SDG 2: Zero hunger, SDG 3: Good health and well‐being

## Abstract

**Objectives:**

Marginalised and non‐white voices are under‐represented in food and nutrition policy in Australia. To achieve equitable food systems that support healthy diets for all, the voices and lived experiences of people from diverse cultural backgrounds must be meaningfully included in decision‐making. This research aimed to understand Sub‐Saharan African (SSA) young people's experiences of food and nutrition and identify opportunities for culturally sensitive policies that can improve these experiences in Victoria, Australia.

**Design:**

A qualitative approach, grounded in critical theory, experience‐based co‐design and the Social and Emotional Wellbeing framework, was employed to attain a policy‐relevant understanding of SSA young people's perspectives and engagement with food and nutrition in Australia. In‐depth interview guides were co‐designed with an advisory committee of multicultural young people, including two young SSA Australians.

**Results:**

Twenty young people (15–25 years) with East and West African backgrounds were interviewed, mostly from metropolitan Victoria (75%). Four overarching themes were identified describing (i) young people's experiences of cultural food shaping their identity but being difficult to access due to systemic barriers, (ii) food affordability and economic opportunities influencing community wellbeing, (iii) the mixed relevance of dominant food and nutrition policy priorities and (iv) the need for stronger government prioritisation of migrants and equity.

**Conclusion:**

This research identifies food as a critical determinant of health and wellbeing among SSA youth, serving not only as a cultural factor but also as a pathway for economic and social advancement. Mainstream food and nutrition policies do not fully address the priorities of SSA youth and opportunities exist to develop more culturally sensitive policy options. Additional efforts are required from the public health community to address discrimination across food systems, starting with better inclusion of multicultural experiences and voices in research, policy and practice.

## 1 Introduction

Inequalities in nutrition, diet‐related health and food security are observed globally across multiple measures of social position, including age, ethnicity, income, education, gender and occupation [1]. Although socioeconomic inequalities have received considerable research attention, ethnic and racial inequalities in food insecurity and nutrition research remain lesser explored, particularly across many high‐income countries [2]. Existing evidence from Australia, Canada, the United Kingdom (UK) and the United States (US) highlights significant ethnic and racial inequalities in some diet‐related health outcomes, such as a higher obesity prevalence among children and young adults [3, 4, 5, 6]. For example, the US shows particularly stark inequalities, with an obesity prevalence of 24% among non‐Hispanic Black children, 26% among Hispanic children and 27% among Mexican American children, compared to 16% among non‐Hispanic White children in 2017–18 [5]. Similar trends have been observed in the UK, where Black African children (4–5 and 10–11 years) experienced the highest prevalence of overweight or obesity at 48%, compared to 35% among White British children in 2022–2023 [6]. Food insecurity is also high and inequitably distributed among young people. A national cohort study from Canada indicated that 30% of young people lived in food‐insecure households in 2016, with 1.96 and 4.25 times higher odds of moderate and severe food insecurity among Black or Indigenous young people compared to young people from other ethnic backgrounds [7].

Australia is one of the most multicultural countries in the world, with approximately half of its population born overseas or having a parent born overseas [8]. Although inequalities in obesity among children are often measured based on Aboriginal and Torres Strait Islander status, other ethnic, racial or cultural differences in diet‐related health are often not included in national monitoring [9]. To bridge this evidence gap, a study conducted in 2014 among children 9–12 years (*n* = 2407) in Victoria found that South Asian (52%), Middle‐Eastern (55%), African (57%) and Southeast Asian (58%) children were more likely to consume fast foods weekly compared to non‐culturally diverse children (43%) [3]. Additionally, Middle‐Eastern children were found to have significantly higher odds of experiencing obesity compared to their non‐culturally diverse peers, with differences according to other ethnicities being non‐significant [3]. Yet, studies exploring in‐depth food and nutrition experiences among culturally diverse young people in Australia are limited in their scope and/or outdated (conducted approximately a decade ago) [10].

The number of people with African migrant backgrounds in Australia is increasing [11]. In 2023, there were 437 710 Sub‐Saharan African (SSA) migrants (1.6% of the Australian population), increasing by almost 200% from 146 230 in 2000 [11]. These figures exclude individuals of African descent from countries like the UK, New Zealand and the US, which diversifies Australia's African‐heritage communities. Including these groups significantly increases the number of Australians with African ancestry, highlighting the increasing importance of this demographic in food and nutrition policy discourse [12, 13].

Young people who identify as SSA in Australia (herein referred to as young SSAs or SSA young people) constitute a culturally diverse and rich cohort. However, this group faces significant challenges due to mainstream discourse, policy and services that can create a pervasive sense of otherness and guilt, as well as creating significant discrimination and disrupting a sense of belonging in relationships, employment, education, health services and housing [12, 13]. A 2020 study found that SSA young people aged 15–24 years were up to 10 times more likely than Australian‐born young people to develop a psychological disorder [14]. Young SSA's unique experiences of multiple forms of discrimination are likely to influence their unique food and nutrition experiences. Consistent with diet acculturation theory [15], qualitative evidence suggests that young African refugees have often adopted Western diets not merely out of preference and convenience, but as a response to structural barriers and existing food environments [10]. This shift can occur to fit in with peers at school and is influenced by altered family roles and decreased home cooking, highlighting that these dietary changes are often driven by environmental and socioeconomic circumstances rather than individual choice [10]. Consequently, Westernised dietary changes can lead to adverse health impacts, with maintenance of traditional cultural practices shown to be associated with protective traits such as a lower prevalence of obesity and sedentary behaviours among SSA children in Victoria, Australia [16].

Inadequate inclusion of the voices of people who experience marginalisation in food and nutrition policy—which seeks to advance food security and diet‐related health across populations but is rooted in colonial histories—continues to hinder meaningful progress towards culturally sensitive approaches [17]. Achieving healthy, sustainable and equitable food and nutrition outcomes for all will require the meaningful inclusion and representation of people of colour and people from diverse social, cultural and economic positions [18]. This study aimed to contribute evidence on this topic in the Australian context by advancing our understanding of the policy‐relevant food and nutrition experiences of SSA young people in Victoria.

We aimed to address the following research questions:

1. What are the policy‐relevant lived experiences of food and nutrition among SSA young people in Australia?
2. How do these experiences differ between metropolitan and regional communities?
3. What social, cultural and political factors enable or constrain SSA young people in Australia from achieving their ideal food and nutrition experiences?

## 2 Methods

In this study, SSA comprises all African countries and territories located fully or partially south of the Sahara Desert. This definition aligns with the geographic classifications of the Australian Bureau of Statistics and the United Nations. Consequently, it excludes North African countries such as Algeria, Egypt, Libya, Morocco, Sudan and Tunisia [8]. All participants in the study had their origins (either by birth or by parental birth) in this region [8].

### 2.1 Study Design and Theoretical Frameworks

A qualitative approach, grounded in critical theory, was employed to attain a comprehensive understanding of SSA young people's food and nutrition experiences and policy perspectives in Australia. We report our study according to the COnsolidated criteria for REporting Qualitative research (COREQ) checklist (Table A1) [19]. Additionally, we used two frameworks to ensure the research was conducted in a culturally safe, self‐determined and meaningful way.

Firstly, experience‐based co‐design [20] informed the inclusion of community members with relevant lived experience across the project lifecycle, an approach previously used to re‐design mental health systems with children and young people [20]. For this study, an advisory committee of three young multicultural Australians was established: Two male African Australians (one from East Africa, one from West Africa) and one female Filipino Australian. The African Australian members held primary decision‐making power regarding the cultural appropriateness of recruitment materials and interview questions, whereas the Filipino Australian provided insights on shared issues of cultural food access and policy exclusion among migrant‐background youth in Australia. All interviews were conducted and analysed by a SSA Australian, ensuring that data collection and analysis remained grounded in SSA lived experience.

In addition, the Social and Emotional Health and Wellbeing Framework [21], was used to explicitly recognise how people from diverse cultures have unique experiences of wellbeing. Although originally developed with a focus on the wellbeing of First Nations communities in Australia, our advisory committee identified that traditional Western frameworks had limited application to understanding SSA young people's experiences of food and nutrition, as they do not fully address how individual health is explicitly linked with family, community, country and systems of discrimination. The Social and Emotional Health and Wellbeing Framework comprises nine underlying principles describing health as holistic; the right to self‐determination; the need for cultural understanding; the impact of history in trauma and loss; recognition of human rights; the impact of racism and stigma; the importance of family, kinship and community; cultural diversity and continuity; and recognition of strengths [21]. These principles were used to inform our interview question topics.

### 2.2 Data Collection

Whilst our advisory committee co‐designed multiple data collection methods (e.g., interviews, photovoice, focus groups, written autoethnographies) to enable SSA young people to have choice in how they participated in the study, all participants opted for an in‐depth individual or group interview. The semi‐structured interview guide included 17 questions and was piloted with two SSA Australian young people and revised to improve accessibility (final version available in Appendix B). Questions related to the perceived accessibility and healthiness of cultural foods; role of food in the African community; food‐related health challenges in the community; experiences of racism in food settings; cultural relevance of common food and nutrition policy recommendations; and top policy recommendations.

All interviews were conducted by the lead researcher KM, a 19‐year‐old SSA Australian, who was trained by CZ, an experienced qualitative, nutrition and health equity postdoctoral researcher. This enabled power imbalances with participants to be minimised. Whilst participants may have previously seen the researchers talk about the work, no participants had prior relationships with the research team. Participants had the option to take part in an interview online or in person at a community centre, which was audio‐recorded via Zoom. Interviews were 30 to 60 min in duration.

### 2.3 Sampling and Recruitment

We aimed to recruit 20 young SSAs to participate in the study. This sample size has been shown to be sufficient to attain data saturation in qualitative research [22]. We also aimed to sample an equal representation of female and male participants. Our sample included participants from East and West Africa and we acknowledge the absence of participants from Central or Southern Africa as a limitation. To be included in this study, participants were required to be: between the ages of 15–30; born or have at least one parent born in a Sub‐Saharan African country and now permanently residing in Australia; willing to discuss their lived experiences of food systems and policy priorities for equitably improving population diets. Additionally, we recognise that migration pathways (e.g., refugee, family, skilled migrant) were not systematically recorded in this study, which is also a limitation. However, based on Australia's migration history and common resettlement patterns, it is plausible that East African participants (Somalia, South Sudan) more often have humanitarian/refugee backgrounds, whereas West African participants (Nigeria, Togo) may more frequently come via family and/or skilled migration pathways [9]. This contextual information is provided to assist in interpreting the findings.

Participants were purposively sampled from networks of the youth advisory committee. These included: university peers, youth services near public housing and African or multicultural community initiatives and groups. Potential participants were approached in person, by email or phone and it was made clear (verbally and through written information) that they were not obliged to participate.

Potential participants were screened and excluded if they were below the age of 15 or experiencing severe mental health challenges. All participants completed the Kessler 10 Psychological Distress Scale (10 short questions on 5‐point scales) [23]. If young people scored highly (≥ 20), any potentially distressing questions about challenges with accessing food were removed from the interview.

### 2.4 Ethical Considerations

The study was approved by the Deakin University Ethics Committee (ID 2023–022). All participants were provided with a Plain Language Statement and Consent Form and written and verbal consent were obtained from every participant. If participants were below the age of 18, parental or guardian consent were obtained to conduct interviews, with two researchers (KM and CZ) present during interviews. All protocols were reviewed by a psychologist (HJ) and public health practitioners (NM, KK) with experience in trauma‐informed care and/or cultural safety to further ensure culturally diverse perspectives could be safely supported during the project. To mitigate any potential discomfort (which was not reported in our study), we focused on creating positive interactions with young people, maintaining equal relationships between the researchers and participants that is by engaging a peer facilitator (KM), and providing flexibility in method, location and time of interview. Community members were renumerated at a rate of $50/h for participation in advisory meetings and interviews.

### 2.5 Data Analysis

The interview recordings were transcribed and inductively coded. Aligning with Braun & Clarke's recommendations for reflexive thematic analysis [24], KM and CZ met regularly to discuss their interpretation of the data, coding, reflections and to create meaningful themes from the codes. Initial coding was conducted using NVivo 14 [25]. Instead of returning participant transcripts (to avoid any potential for confusion, stress or burden), KM led the coding and thematic analysis to ensure themes regarding food, nutrition and policy perceptions were conveyed through the lens of a young SSA Australian, with CZ providing training and supervision. In the event of discrepancy, codes were discussed until consensus was reached.

### 2.6 Positionality Statement

This project stemmed from collaborative work being undertaken by our culturally diverse research team. We collectively identified the systematic underrepresentation of the experiences and voices of communities of colour, particularly African young people, in Australian food policy, research and practice. CZ (a White Greek Australian female) co‐designed and co‐led the research with an advisory committee of three young multicultural Australians (two male African Australians, one female Filipino Australian), with one young African Australian (KM) employed to co‐lead the project. The team met regularly to iteratively adapt our approach and challenge White dominance in public health nutrition practices and policy recommendations.

## 3 Results

### 3.1 Participant Characteristics

Twenty participants completed interviews between August 2023 and March 2024 (see Table **1** Participants characteristics). Fourteen participants were female and six were male. Five participants were between the ages of 16–18, six were between 19 and 21 and the remaining nine were between 22 and 25. The majority of SSA young people had family origins from East Africa, with two from West Africa. One quarter of participants were living in regional areas, with the remainder in metropolitan areas of Victoria. The four participants from regional areas participated in two group interviews.

**TABLE 1:** Sociodemographic characteristics of Sub‐Saharan African Australian youth participants (*n* = 20).

Sociodemographic characteristic	Categories	*n* (%)
Age	15–18 years	5 (25%)
	19–21 years	6 (30%)
	22–25 years	9 (45%)
Gender	Female	14 (70%)
	Male	6 (30%)
Location in Victoria, Australia	Metropolitan Melbourne	15 (75%)
	Regional Victoria	5 (25%)
African region of family origin	East Africa (Somalia, South Sudan)	18 (90%)
	West Africa (Nigeria, Togo)	2 (10%)

### 3.2 Thematic Overview

**FIGURE 1:**
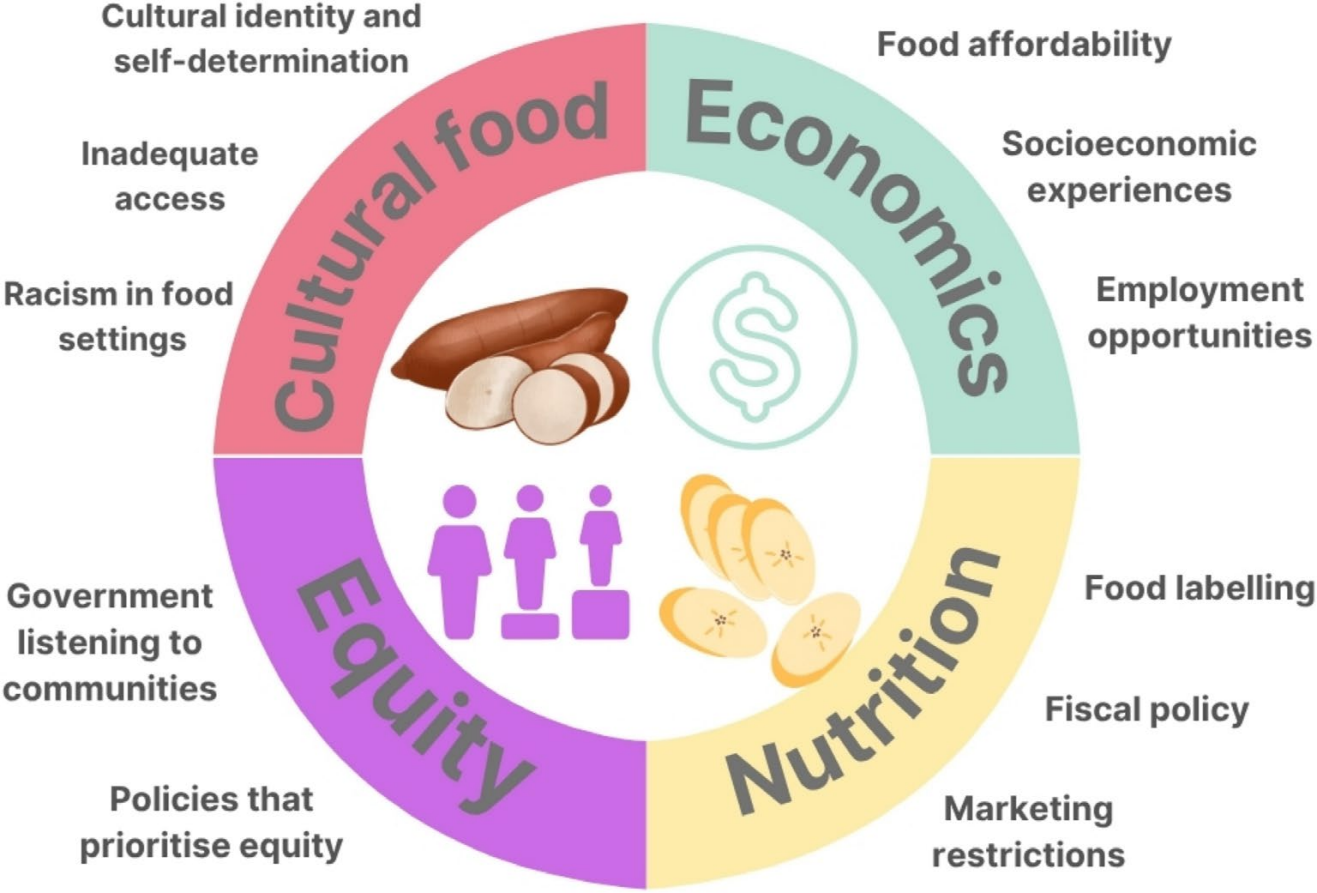
Summary of the policy‐relevant experiences of food and nutrition among Sub‐Saharan African (SSA) Australian young people.

We identified four major themes that described SSA young people's experiences of: cultural food shaping their identity but being difficult to access due to systemic barriers (theme 1), food affordability and economic opportunities influencing community wellbeing (theme 2), the varied relevance of dominant food and nutrition policy (theme 3), and the need for stronger government prioritisation of migrants and equity (theme 4). Figure 1 provides a visual summary of these policy‐relevant experiences and themes.

### 3.3 Theme 1: Cultural Food Shapes Identity but Systemic Barriers to Access Persist

#### 3.3.1 Cultural Food as a Vehicle for Identity, Belonging and Self‐Determination

Participants collectively identified the importance of cultural food for celebrating cultural identity, maintaining connection to culture and developing a sense of belonging. Cultural food was described as a unifying force and the focal point of social gatherings. Young people spoke about how cultural food often involved sharing a plate and how this was an avenue for conversations and social connection between community and family members. Most participants highlighted how African cultural food showcases the strength, spirit and diversity of African culture:

The type of food we eat as well, it's shared. So like you'll have a big bowl and then multiple people will eat at the same time. It's a sense of community as well when you're eating with other people. (Participant 1)

#### 3.3.2 Gendered and Intergenerational Dynamics in Learning Cultural Food Practices

Young people spoke about the importance of intergenerational sharing of cultural knowledge via the creation of cultural dishes at home. It was common for parents to have taken, or be willing to take, time to share cooking knowledge and recipes with their children to preserve their cultural heritage. However, when young people were asked about their interest in learning to create their cultural food, they reflected on the need to balance their changing responsibilities at home and outside of home. Some participants thought it was necessary to learn how to cook their cultural foods because they would be required to make dishes for their younger siblings. Other participants described having no imminent need to learn how to cook cultural foods for themselves or their family as they had other roles and competing priorities such as school and work. To some extent, it was expressed how there was a greater responsibility for females than males to learn how to cook cultural foods. According to one participant:

My mum definitely wants us learning. She makes a lot of effort to transfer those skills because her mum did the same to her… if they are the eldest daughter and they do need to take on that responsibility to cook the food when their mum's not there, they are more receptive to learn…I am not that receptive because I don't really have the responsibility to cook for anyone else but myself. (Participant 3)

The concept of ‘keeping culture alive’ was referenced multiple times by participants in relation to their experiences of food. Cultural food was viewed as a mechanism to foster self‐determination via having agency and control over a positive and empowering connection to culture. Many participants mentioned that African‐owned food businesses were spaces for fostering a sense of belonging and enhancing connections to cultural identity, for example, by hosting community events. These businesses were seen as avenues to showcase the diversity of African culture through food and provided opportunities for cross‐cultural exchange and understanding:

African restaurants actually play, I believe, a pivotal role in forming an identity for us in Melbourne and in Australia. It advertises the fact that we exist as… more than just units on a spreadsheet, we have something to contribute that other Australians can also enjoy and participate in. (Participant 6)

Despite the significance of African cultural food, most participants expressed a risk of losing elements of their cultural identity due the inaccessibility and lack of representation of their cultural foods in Western foodscapes.

#### 3.3.3 Inadequate Access to Cultural Foods and Ingredients

It's sad because moving to another country has led to a loss of so many things. But the biggest one, of course, is language and food. (Participant 3)

Most participants reflected on the barriers to accessing cultural foods and ingredients. All participants stated that there was a dearth of African cultural food and ingredients in dominant chain supermarkets, such as Woolworths and Coles. Cultural food and ingredients were often purchased at small African grocers. However, participants discussed how these African grocers were concentrated within specific geographical localities. As participants did not reside in these areas, most spoke of the inconvenience of accessing cultural foods and ingredients. Many young people from urban areas reported how they or their family members would often drive or take public transport for more than 25 min to reach these grocers. In regional areas, participants described travelling more than 4 h to purchase cultural foods in the closest major city. In the absence of African grocers, a few participants described accessing Indian grocers to purchase alike replacement ingredients, but families could not shop there to purchase all their cultural foods.

Similarly, all participants noted that African‐owned restaurants were scarce in both their local neighbourhoods and in metropolitan and regional Victoria more broadly. Some participants observed ‘not having enough diversity with other cultural foods in shopping centers’ (Participant 14) and the inadequate representation of African restaurants in the food retail market compared to other cultural foods.

#### 3.3.4 Experiences of Racism in Food Related Settings

Many young people expressed concerning experiences of racism, including in Australia's dominant chain supermarkets. Their experiences of racial profiling, unwarranted suspicion, watching and following of African young people by security in supermarkets led to negative self‐worth and erosion of dignity. One participant explicitly noted, ‘there is racial profiling all the time at my local (supermarket name)’ (Participant 4). Although experiences of racism in supermarkets were reported as common, young people were unclear about how it affected their food purchases.

Additionally, stigmatisation of cultural foods in school and work settings was highlighted. However, increased awareness and acceptability of diverse cultural foods over time in primary and secondary school settings were thought to have increased young people's comfort in bringing cultural food to school. In workplace settings, a few participants expressed hesitation to bring cultural food due to the risk of embarrassment from coworkers judging its appearance and smell. Despite this, many participants stated that they were ‘very proud’ of their cultural foods and that this pride superseded concerns of experiencing stigma in these settings.

### 3.4 Theme 2: Food Affordability and Economic Opportunities Influence Community Wellbeing

#### 3.4.1 Food Cost and Affordability

Rises in food prices over recent years were discussed by multiple study participants, who indicated that many African families could not afford to purchase healthy food because it is too expensive. As a result, some young people stated that parents resorted to purchasing cheaper foods that are typically more energy‐dense and low‐nutritional value. Against a backdrop of a cost‐of‐living crisis in Australia, prioritising healthy food was even more difficult for larger families. One young person summarised this:

It's healthy, but we can't afford because it's too expensive. They tend to buy all the cheap ones and then to make their meals according to that. Especially in the African community or family, they tend to have a lot of kids in the household, which it becomes more expensive for the parents to prioritise healthy food than unhealthy food. (Participant 12)

Some participants also suggested that cultural foods at African grocers were ‘way more expensive than like it usually is (in Africa)’ (Participant 1). Trade barriers to importing cultural foods were noted by young people as a key reason for this.

#### 3.4.2 Socioeconomic Hardship and Social Supports

Some participants perceived food insecurity, due to socioeconomic hardship, to be a significant issue in African communities, especially in the cost‐of‐living crisis. Other young people were less sure of the level of food security within the community. From one young person's experience:

I lived in the commission flats, so I definitely saw from that end, it was like I could see people struggling and not being able to afford food. (Participant 4)

Participants described how the African community provides support to families and individuals experiencing hardship and food insecurity. One participant highlighted the role of community leaders and organisations, referring to their leadership in distributing food hampers for community members in need during the COVID‐19 lockdowns. Whilst some young people indicated that leaning on community for support is a cultural strength, other participants indicated that there can also be stigma associated with seeking support. According to one participant:

It's family honour, it's personal, saving face. A lot of the same issues that I think affect a lot of micro communities, which is that to ask for help is to bring shame upon your family and is to say that your family cannot provide for you or that your family is lacking in some way. (Participant 6)

#### 3.4.3 Supporting African‐Owned Food Businesses and Better Employment Opportunities Across Food Sectors

In addition to being spaces for community to engage with and explore their culture, participants perceived African owned food businesses as an opportunity to support community development. African owned food businesses were recognised as pathways to drive economic prosperity in community, especially among aunties in the community. Nevertheless, participants described barriers associated with starting up a food business and receiving adequate supports to do so. Participants highlighted the lack of targeted grants for starting up small cultural food businesses as well as the lack of ongoing support for those that had been established. Some young people indicated that while grants may exist for small businesses, the application processes are generally complex and promotion of these grant opportunities is limited across African communities. Participants discussed the need to provide more supports with grant applications, including improving community engagement to overcome language barriers and address community queries. Young people recommended strengthening economic supports and plans to improve the long‐term sustainability African owned food businesses:

I feel like there's not enough funding to the community for them to genuinely have a shop and actually keep it up to the standards and stuff. (Participant 11)

On the topic of employment, some participants described how one of the first lines of employment for many young people, notably African young people, was fast‐food chains. Contributing to this were factors such as convenience and proximity, reliability of shifts, standard pay and the social environment in these settings. Young people perceived that opportunities for employment outside of fast‐food chains tended to be limited for people who were in high school. There was little discussion around how working in fast‐food chains influenced young people's eating choices. One participant explained how:

Fast food outlets are really everywhere, so it's very easy to find the job one that's really close to you, so you don't have to travel far for work. They also pay pretty well compared to some of their working for a privately‐owned organisation. (Participant 15)

### 3.5 Theme 3: Dominant Food and Nutrition Policies Vary in Relevance for African Australian Youth

Participant's perceptions of dominant food policy recommendations were mixed. Some participants thought that these policies could help African young people and the broader community achieve healthier diets and others thought they would not make a difference to community health.

#### 3.5.1 Food Labelling

Whilst many participants saw the value of food labelling (any type e.g., Nutrition Information panels and Health Star Ratings) to facilitate healthier choices, it was often thought to have complex language. Some participants expressed how food labels posed language barriers for newly arrived and resettled migrants. Participants also indicated that their interpretation of food labels was limited as they did not learn about food labelling in school, even though they may have learnt about nutrition broadly. According to one young person:

I would say no [food labelling is not helpful], because labels are typically very convoluted and they've got a lot of writing in them. I think that's also expanded by the fact that adequate health information isn't given to children when they're in school or even to adults when they migrate here. (Participant 15)

#### 3.5.2 Food and Beverage Fiscal Policies

Whilst some participants suggested that sugary drink taxes could deter purchasing and therefore promote healthier choices, others suggested that taxes may not be the most effective tool as young people will still purchase sugary drinks if they are motivated to. As such, participants often called for greater education to promote healthier lifestyles. Further, multiple participants expressed concern over water being more expensive than sugary drinks in many settings:

I reckon water should be a bit cheaper. (Participant 14)

#### 3.5.3 Unhealthy Food Marketing Restrictions

Most participants agreed that government intervention on unhealthy food marketing across settings would support healthier diets for African young people. Some participants spoke to unhealthy food marketing being disproportionately targeted to population groups in low socio‐economic positions, highlighting that these groups generally have lower education levels and may be more susceptible to having their food choices influenced by unhealthy food marketing. This is summarised by one participant below:

it's really important to advertise the drawbacks of eating unhealthily, especially when in some areas there is an increase in advertising for junk foods by these large corporations. (Participant 6)

Conversely, a few participants opposed the idea of unhealthy food marketing restrictions, suggesting that a balance of healthy and unhealthy food is good for people's diets and that corporations should have the freedom to promote their own businesses and brands, with guidelines to limit marketing to children and young people.

### 3.6 Theme 4: The Need for Stronger Government Prioritisation of Migrants and Equity

#### 3.6.1 Willingness of Government to Listen and Commit to Equitable Actions

Most participants highlighted that governments in Australia do not sufficiently listen to or support African communities. African young people expressed that they typically did not have opportunities to have their voices heard by decision makers. Some participants mentioned that governments do not engage African young people and the broader African community in decisions that affect them, especially those around food and nutrition. One participant stated, ‘I would say that governments…don't care of the opinions of Africans’ (Participant 11).

An example of how a lack of government proper community engagement impacted their food and nutrition experiences was the snap COVID‐19 lockdown of the young people's local public housing towers in 2020 [26]. Multiple participants highlighted that during this time many African families struggled to receive essential supplies and staple food items, let alone any culturally appropriate food. One participant stated that families ‘were given boxes of food that I think some of them were expired or mouldy’ (Participant 10).

As such, young people inferred that this neglect for the community's sense of autonomy and dignity could have been prevented with genuine and ongoing government commitment to understanding the needs and priorities of public housing tower residents, including those from migrant communities. As previously mentioned, another example of how young people conceptualised a lack of government commitment to supporting the wellbeing of the African community was the perceived lack of government commitment to support African‐owned food businesses.

#### 3.6.2 Young People's Policy Priority Areas

In response to being asked what food and nutrition policy actions young people would implement if they were in a policy leadership position (i.e., prime minister), a diverse range of policy options were stated. Policy recommendations were relevant across all themes and have been summarised in Table 2. African young people's policy priorities included working towards addressing the drivers of systemic inequalities related to food and nutrition, greater prioritisation of equity across all government policy and creating environments that promote healthy diets and facilitate fair access to affordable and culturally appropriate food. According to one participant who outlined the overarching importance of equitable policy processes:

I don't think the government has done a great job with representation, and perhaps that's where we start, because if you have a diverse group of Australians, they can better create policy… I think liaising with people and with community organisations that really do good work in terms of bringing the community together is also a great place to start because, again, they have access to a lot of African people… so it's more than just a cosmetic shift. (Participant 6)

**TABLE 2:** Sub‐Saharan African (SSA) young people's policy priorities to improve their experiences of food and nutrition in Australia.

*Cultural food shapes identity but systemic barriers to access persist*	
1	Include African cultural foods and ingredients into chain supermarkets
2	Make fewer gelatine containing products (for Muslim community members)
3	Diversify staff in supermarkets

*Food affordability and economic opportunities influence community wellbeing*	
4	Introduce an equity‐driven monthly food stipend for families experiencing disadvantage
5	Greater funding for African owned food businesses, including grocery stores and restaurants
6	Reduce trade/import barriers for cultural food and ingredients
7	Implement business jumpstart programs for African women and girls
8	Reduce grant access barriers
9	Introduce an equity driven monthly food stipend for families experiencing disadvantage
10	Implement price caps for healthy foods and beverages

*Dominant food and nutrition policies vary in relevance for African Australian youth*	
11	More culturally appropriate and easy‐to‐understand food labelling, including better regulating nutrition labels on imported foods
12	Greater educational content around healthy food that is culturally appropriate
13	Implement programs that support community to understand the link between mental health and food
14	Greater regulation of harmful industries, including junk food marketing
15	Implement food and grocery voucher programs for newly arrived migrants
16	Mandate cooking classes in schools
17	Targeted marketing campaigns to promote the nutrition benefits of different foods
18	Restrict development of fast‐food chains in built neighbourhoods

*Stronger government prioritisation of migrants and equity*	
19	Better and more ethical engagement with minority groups
20	Better housing standards

Policy recommendations also involved supporting the creation of more African food businesses, improving engagement pathways between government stakeholders and community, raising housing standards, making nutrition education in schools more culturally appropriate and greater regulation of harmful marketing by unhealthy food industries.

## 4 Discussion

This study provides critical insights into the experiences of SSA young people regarding food and nutrition, identifying culturally sensitive policy opportunities to enhance these experiences in Australia. Although SSA migrants are considerably diverse in terms of geographic origins, language and traditional food practices, they share fundamental cultural commonalities rooted in collectivist values emphasising interconnectedness, strong family units and community cohesion, often reinforced by shared history, tribal and religious traditions [27]. Despite these cultural strengths, SSA young people collectively face systemic challenges in host countries, such as policy landscapes that seldom seek to accommodate their cultural food practices, nutritional needs and economic wellbeing [27]. Grounded in critical theory and participatory frameworks, our findings illustrate how these structural inequities undermine food security, cultural continuity and health equity for SSA young people in Australia.

### 4.1 Shared and Distinctive Experiences of SSA Youth

A key question is whether the experiences described here are unique to SSA youth or reflect broader patterns among migrant and ethnic minority groups in Australia. Many findings mirror challenges faced by other migrant communities, such as limited access to cultural foods, language barriers in food labelling, financial constraints on healthy eating and balancing traditional food practices with Western environments. However, some issues stand out for SSA youth. Notably, racial profiling and surveillance in supermarkets, reported by several participants, highlight specific anti‐African racism in Australia [21]. The importance of African‐owned food businesses as spaces for cultural identity, community self‐determination and economic resistance was also particularly prominent. In addition, communal food practices like shared plates and extended family food networks may be more deeply rooted in SSA traditions than in some other migrant groups. These differences matter for how food policies define ‘healthy eating.’ A direct comparative study is needed to clarify which experiences are shared and which are unique to this group. With these patterns in mind, we now examine the central role of traditional food in shaping cultural identity among SSA youth.

### 4.2 Traditional Food and Cultural Identity

Traditional food practices are central to how SSA young people experience and express cultural identity in Australia. An intersectional lens is essential for understanding the food and nutrition experiences of SSA young people. Factors such as gender, migration status, race, socioeconomic status, religion, generational background and region of origin interact with each other to influence food access, preparation and perceptions. Our findings highlight how these intersections manifest in reality. For example, young women may face gendered expectations to learn cultural cooking, which young men did not experience, illustrating the intersection of gender and cultural preservation norms. Additionally, socioeconomic status intersected with geography: participants from public housing and regional areas reported the most significant barriers to accessing cultural foods. Migration history also plays a crucial role; whether families arrived as refugees, through skilled migration, or were born in Australia, it likely affects their economic resources, social networks and familiarity with Australian food systems. However, our sample size limited our ability to break down these experiences by specific migration pathways.

Our findings also show how the maintenance of traditional food practices, including preparing cultural meals and communal eating, serves as acts of resistance against systemic exclusion and are key for preserving cultural identity and intergenerational knowledge [28]. SSA young people's narratives are consistent with findings from Myanmar refugees in Australia and Indian migrants in the Netherlands, where shared meals strengthen community networks and belonging [29, 30]. Despite young people describing the importance of food and language for cultural identity and belonging, our study also supports the need to further consider intersectional differences in food and nutrition experiences [31]. Other research has described how young women are often disproportionately responsible for maintaining food traditions [32]. Similar findings have been reported among various migrant cultures, including Afghan women in Australia and Arabic and South Asian women in Canada [33]. Moreover, a 2015 mixed methods study of migrants living in regional areas of Tasmania, Australia, found that gender (being female), socioeconomic position, place of birth, duration post‐migration, English proficiency and education levels were all key determinants of food insecurity [34]. Although our findings reflect the perspectives of predominantly young women from diverse SSA young people's backgrounds, many of whom lived in metropolitan public housing in Victoria, more explicit inquiry into the intersections between gender, race, socioeconomic status and migration status in shaping dietary and nutrition experiences could help inform gender‐transformative policy priorities—aligning with global agendas [35].

### 4.3 Addressing Structural Barriers to Accessing Culturally Appropriate Healthy Food

Our study highlighted multiple structural barriers that compromised the role of traditional foods in preserving cultural identity among SSA populations in Australia, including through the racialised geographic access to traditional foods. The concentration of African grocers in specific suburbs and the absence of cultural ingredients and foods from mainstream supermarkets could point towards issues of structural and spatial racism. To this extent, migrant communities are forced to undertake extended travel for essential cultural ingredients due to underinvestment in providing essential community infrastructure [36]. Geographical marginalisation can also accelerate assimilation to Western food environments and thus diets [37]. This issue is particularly key in regional Australian areas, where governments often settle migrants to decentralise population growth and address labour shortages [38, 39], but fail to provide access to basic needs, including access to cultural foods. Whilst government food security policies recognise the importance of supporting migrant and refugee food access in Australia [40], policy options are often individualised (e.g., literacy and food relief programs) rather than focused on building culturally appropriate food infrastructure. There is also a well‐established problem with emergency food relief programs seldom providing culturally appropriate foods [41].

The findings regarding the economic determinants of food and nutrition among SSA young people and their communities included ideas related to the high cost and unaffordability of healthy and cultural foods, socioeconomic hardship and social supports. Similar findings were observed in a scoping review of 68 articles examining how migrants experienced food environments in high‐income countries [41]. These dynamics are particularly pronounced for communities with lower paying jobs or in larger families, where the financial burden of buying food can be magnified. It is also exacerbated by the cost‐of‐living crisis, trade barriers to importing cultural foods and fast foods being perceived to be cheaper than cultural foods [41]. Furthermore, scholars have argued that racism underpins the stratification of food security and nutrition outcomes due to the intersection between socioeconomic position and race [42].

The potential of African‐owned food businesses to foster community cohesion, communal eating, food security as well as economic prosperity was discussed by SSA young people. However, participants described barriers to establishing and sustaining food businesses in their communities, underpinned by systemic exclusion from grant schemes and economic opportunities. These findings are consistent with the application of critical race theory to food systems, which posits migrant communities are often excluded from equitable economic participation and are often used as cheap labour (e.g., in agriculture and fast food chains as first lines of employment in Australia) [43, 44]. Decision makers need to provide better targeted economic supports for migrant communities, informed by genuine community engagement, with a view to concurrently support local economic development and better access to healthy cultural foods.

### 4.4 Food and Nutrition Policy Relevance for Culturally Diverse Migrants

Evidence suggests that many diverse cultural diets, including traditional African ones, support better diet‐related health than Western alternatives [45]. Recognising this, Burt's analysis stated:

Ultimately, promoting the (Mediterranean Diet) as a gold standard marginalizes people from non‐white cultures by maintaining White culture as normative. In order to better serve and include Black, Indigenous, and People of Color, dietary recommendations need to become as diverse as the US population. Doing so will also improve cultural humility among professionals, beget anti‐racist dietary research, and promote a more evidence‐based dietary perspective.(Burt, Journal of Critical Dietetics, Vol 5 [2, p. 41], [44])

The perceived limitations of mainstream food policies for SSA youth suggest that current policy instruments may fail to account for the cultural, linguistic and socioeconomic realities of many migrant communities in Australia. Interestingly, a 2018 cross‐sectional study found that minority ethnic groups in five countries tended to understand nutrition information panels more than majority ethnic groups [46]. No differences were observed between the groups for front‐of‐pack label use [46]. However, few studies have verified these differences and focused on investigating what meaningful front and back‐of‐pack labelling systems look like for culturally diverse communities, including accounting for barriers such as language proficiency, health literacy, education levels, consumer distrust and relevance for cultural food practices.

Other evidence from the USA has shown that racial differences exist in parental support for taxation and marketing restriction policies to reduce sugary drink consumption among children [47]. Black and Latinx parents were more likely to support all arguments for a sugary drink tax than White parents, and also supported marketing restrictions more when arguments were made to address racial targeting by industry actors [47]. Our participants generally showed support for these policies but also reflected on how they would not sufficiently address structural barriers to nutrition that migrant youth face. SSA young people's observation about water affordability also points to a critical commodification failure where healthy options can become luxury goods for communities facing financial hardship.

Overall, the lack of recent food and nutrition evidence examining inequalities according to race or ethnicity is problematic. In Australia, there is no routine monitoring of food insecurity and diet patterns among migrants, limiting policymakers' ability to identify and address food and nutrition challenges. Future research should prioritise systematic data collection to inform effective interventions and help practitioners develop better critical consciousness. Additionally, more research is needed to increase understanding of the impacts of racism and discrimination on food and nutrition outcomes among African and other migrant communities.

### 4.5 Self‐Determined Food and Nutrition Policy

The COVID‐19 public housing tower lockdowns that SSA young people described, including the distribution of expired food, illustrate how government responses can exacerbate food insecurity and undermine community dignity [26]. This incident highlights the importance of establishing trust and open lines of communication to ensure that policies are responsive to the needs of all community members [48]. Participants' recommendations point towards participatory models of food systems governance that prioritise migrant and community knowledge in decision‐making [48]. Their calls for improved community engagement pathways and representative policymaking align with emerging food sovereignty movements globally [49].

In addition, SSA young people's emphasis on developing African‐owned food businesses suggests that investing in local initiatives could help to foster economic resilience, social support, cultural sovereignty and food security in their communities. Further, several participants emphasised the role of community leadership and resourcefulness in overcoming food insecurity, reflecting findings from previous studies among ethnic minority communities in Canada [50]. For example, Somali refugees in Toronto created food‐sharing networks to combat retail redlining, whereas South Asian women in Vancouver established rotating credit systems for ethnic grocery cooperatives [50]. Ultimately, addressing the social determinants of nutrition among SSA youth requires a multifaceted approach that includes improving food policy relevance, enhancing government responsiveness to social exclusion and fostering meaningful community engagement. These findings have several implications for research, policy and practice, which are summarised in Table 3.

**TABLE 3:** Summary of implications for research, policy and practice.

1	Understand the role of traditional diets and foods in improving the wellbeing of migrant communities, including for different intersectional groups such as girls and young women.
2	Increase access to affordable cultural foods in metropolitan and regional communities.
3	Develop more comprehensive policy responses that address structural (i.e., economic, employment, etc.) barriers to improving food security among migrant communities. This will require shifting from a dominant focus on individual policy responses such as emergency food relief and literacy programs.
4	Invest in and evaluate self‐determined community initiatives such as social enterprises or food businesses that can help to achieve economic, social and health benefits for migrant communities.
5	Examine the impacts of mainstream food and nutrition policies among diverse ethnic and racial communities.
6	Systematically monitor food insecurity, nutrition and diet‐related outcomes of diverse migrant communities.
7	Examine the impacts of racism and discrimination in food systems.
8	Foster the leadership and more equitable economic participation of migrant communities across food systems.

### 4.6 Strengths and Limitations

This study contributes to notable recent literature gaps on the food and nutrition experiences of diverse racial and ethnic communities in Australia. The work was co‐led by a young African Australian, with a multicultural advisory committee advising on all aspects. Another critical aspect of the project included convening a youth‐led ‘Bridging Voices Through Food’ community dialogue and preparing a media release to support timely dissemination of the research, amplify youth voices and public advocacy, and connect young people with decision makers from government, non‐government and academia.

There is scope to strengthen the intersectionality lens of future participant and advisory committee recruitment to achieve greater representation and amplify diverse identities within the African diaspora (e.g., African young people with disabilities, living in regional areas, different age groups and from Western, Central and Southern parts of Africa). Linked to this, opportunities exist to identify better ways to engage young African people in food and nutrition research. For example, our interview guide took several iterations before it was accessible to participants, highlighting the need to work more closely with migrant communities in the design of culturally appropriate research methodologies.

Our sample focused only on young people from East and West African backgrounds, not reflecting the experiences of those from Central or Southern Africa. While shared cultural values and experiences of discrimination may exist across SSA, regional differences in food practices and traditions mean these findings cannot be generalised. Future research should sample across all SSA subregions and migration pathways (refugee, skilled migrant, humanitarian entrant, Australian‐born) to better understand how these identities and structures influence food and nutrition outcomes.

## 5 Conclusions

Our study with 20 African young people demonstrates that access to and representation of cultural foods in Australia is crucial for community health and wellbeing. Additionally, we find intersections of structural racism and economic marginalisation that likely drive inequalities in food and nutrition. Policy actions are needed to improve the availability of cultural foods in neighbourhoods and supermarkets, as well as address systemic barriers such as economic and employment opportunities for young people. Strengthening existing social supports for migrant communities experiencing food insecurity is also essential and investment is needed in self‐determined solutions. This will necessitate conscious efforts to develop authentic community relationships, ensuring that the voices of migrant and racialised communities are not only heard but integrated into the decision‐making processes that support better food and nutrition experiences.

## Author Contributions

C.Z. and K.M. led the study conceptualisation, data collection and manuscript preparation. N.M. and K.K. provided input to the study design as advisory committee members. K.B., D.J., V.N., S.S. and H.J. provided input into the study conceptualisation and design, ensuring rigour and cultural and psychological safety of participants. C.A. and A.I. provided additional cultural insights based on their lived experience and helped to finalise the manuscript. All authors provided critical feedback and reviewed manuscript drafts.

## Funding

This research was funded by a Victorian Health Promotion Foundation (VicHealth) Research Fellowship (C.Z.). K.B. is funded by a National Heart Foundation Future Leader Fellowship (106716).

## Conflicts of Interest

The authors declare no conflicts of interest.

## Data Availability

The data that support the findings of this study are available on request from the corresponding author. The data are not publicly available due to privacy or ethical restrictions.

## Appendix A Bridging Voices Through Food: Sub‐Saharan African Young People's Perspectives of Food and Nutrition Policy in Victoria, Australia

**TABLE A1:** Standards for reporting qualitative research checklist.

Criteria number	Topic	Manuscript page#
	*Title and abstract*	
S1	Title	1
S2	Abstract	2
	*Introduction*	
S3	Problem formulation	1–4
S4	Purpose or research question	4
	*Methods*	
S5	Qualitative approach or research paradigm	4–5
S6	Researcher characteristics and reflexivity	8
S7	Context	3
S8	Sampling strategy	6–7
S9	Ethical issues pertaining to human subjects	7
S10	Data collection methods	5–6
S11	Data collection instruments and techniques	5–6, Appendix B
S12	Units of study	9
S13	Data processing	8
S14	Data analysis	8
S15	Techniques to enhance trustworthiness	8
	*Results*/*findings*	
S16	Synthesis and interpretation	9–18
S17	Links to empirical data	9–18
	*Discussion*	
S18	Integration with prior work, implications, transferability, and contribution(s) to the field	18–23
S19	Limitations	23–24
	*Other*	
S20	Conflicts of interest	25
S21	Funding	25

## Appendix B Semi‐Structured Interview Guide to Understand Young African People's Experiences of Food and Nutrition in Australia and Perspectives on How Their Experiences Could be Improved

**Table:**

Number	Questions	Alignment with the Social and emotional wellbeing framework [1]
1	Can you please tell me about your favourite cultural dish?	Health as holistic
2	Do you prepare these dishes? Who prepares the food in your house?	The need for cultural understanding
3	What things make it hard to prepare and access these foods?	Recognition of cultural diversity
		Recognition of the centrality of kinship
4	Do you think African owned restaurants or supermarkets play a role your community? If so, what is this role?	Health as holistic
5	In what ways to you celebrate your culture with food?	The need for cultural understanding
6	How healthy would you consider your culture foods?	Recognition of cultural diversity
7	What role does food play in your health?	Recognition of the centrality of kinship
	*Background information*: We know that governments have a role to play in promoting the health of communities when it comes to food and nutrition policy. Government can do thinks like mandate better labelling on food products, regulate marketing of junk food., tax the unhealthy stuff and subsidise the healthier options so that they are cheaper and affordable. What we don't know is how culturally appropriate some of these policy recommendations are.	Recognition of cultural diversity
8	How well do you believe the cultural preferences and needs of African communities are considered in these food policy processes?	
9	Do you think better food labelling helps African young people make healthier food choices. Why or why not?	
10	What are your thoughts on government stepping up on stopping junk food marketing?	
11	What do you think about government taxing soft drinks?	
12	Do you have any other ideas of policies and programs that would African young people and their families consume healthier foods?	Self‐determination
13	Do you think the government should better support African owned food businesses?	Recognition of strengths
14	What are some of the real strengths of the African community when it comes to food?	The need for cultural understanding
15	How can leaders across society (e.g., government) better understand and respect the diverse cultural needs and preferences of African youth when it comes to food and nutrition?	Recognition of cultural diversity
		Recognition of the centrality of kinship
16	Have you experienced racism in a food related setting before? Do you have any personal experiences of this or have you heard stories about this?	The impact of racism and stigma
		The impact of history in trauma and loss
17	If you were the prime minister of Australia and your job was to help everyone particularly the African community to access healthy, culturally appropriate and affordable foods… what are the top two policies you would put forward?	Self‐determination
		Recognition of strengths
		Recognition of human rights
